# Neuropsychology of sexuality in older adults: bridging gaps in literature and future directions in research

**DOI:** 10.1007/s40520-024-02885-6

**Published:** 2024-11-16

**Authors:** Federica Taccini, Margherita Rossi, Stefania Mannarini, Marina De Rui, Chiara Ceolin, Biancarosa Volpe, Michela Sarlo, Daniela Mapelli, Giuseppe Sergi, Maria Devita

**Affiliations:** 1 Department of Philosophy, Sociology, Education, and Applied Psychology, Section of Applied Psychology, University of Padua, via Venezia, 14, Padua, Italy; 2 Center for Intervention and Research on Family studies-CIRF, Department of Philosophy, Sociology, Education, and Applied Psychology, Section of Applied Psychology, University of Padua, Padua, Italy; 3 Department of General Psychology (DPG), University of Padua, Via Venezia 8, Padua, Italy; 4 Geriatrics Unit, Department of Medicine (DIMED), University of Padua, via Giustiniani, 2, Padua, Italy; 5 Department of Communication Sciences, Humanities and International Studies, University of Urbino Carlo Bo, Via Saffi 15, Urbino, 61029 Italy

**Keywords:** Sexuality, Sexual stimuli, Older adults, Aging, Neuropsychology, Psychophysiology

## Abstract

Sexuality is a fundamental part of human existence and it encompasses thoughts, desires, behaviors, relationships, as well as neuropsychological and physiological components. However, sexuality in older adults is under-researched from the neuropsychological and psychophysiological perspectives and is often neglected by healthcare providers in the clinical practice. This article aims to explore the state of the art on the neuropsychology and psychophysiology of older adults’ sexuality, proposing future research directions and emphasizing its significance. By summarizing current knowledge on the sexuality of younger individuals, it was possible to lay the groundwork for formulating research questions about older adults’ sexuality. The implications proposed in this article will potentially impact both the scientific and also the clinical field. In fact, gaining insights on the neuropsychological and psychophysiological aspects of sexuality in healthy older adults can also shed light into those with neurocognitive disorders.

## Sexuality in older adults

The World Health Organization describes sexuality as a fundamental part of human existence, including aspects such as biological sex, gender identities, sexual orientation, intimacy, and procreation [1]. Sexuality is manifested through thoughts, desires, attitudes, relationships, neuropsychological and physiological aspects which are not necessarily expressed simultaneously. Furthermore, sexuality is shaped by a combination of biological, psychological, neuropsychological, physiological and social influences [1, 2]. This definition applies universally across all ages [2]. Research shows that older adults are sexually active, participating in both genital (e.g., intercourse) and non-genital activities (e.g., kissing), and engaging in solitary sexual activities (e.g., masturbation) [3].

**Fig. 1:**
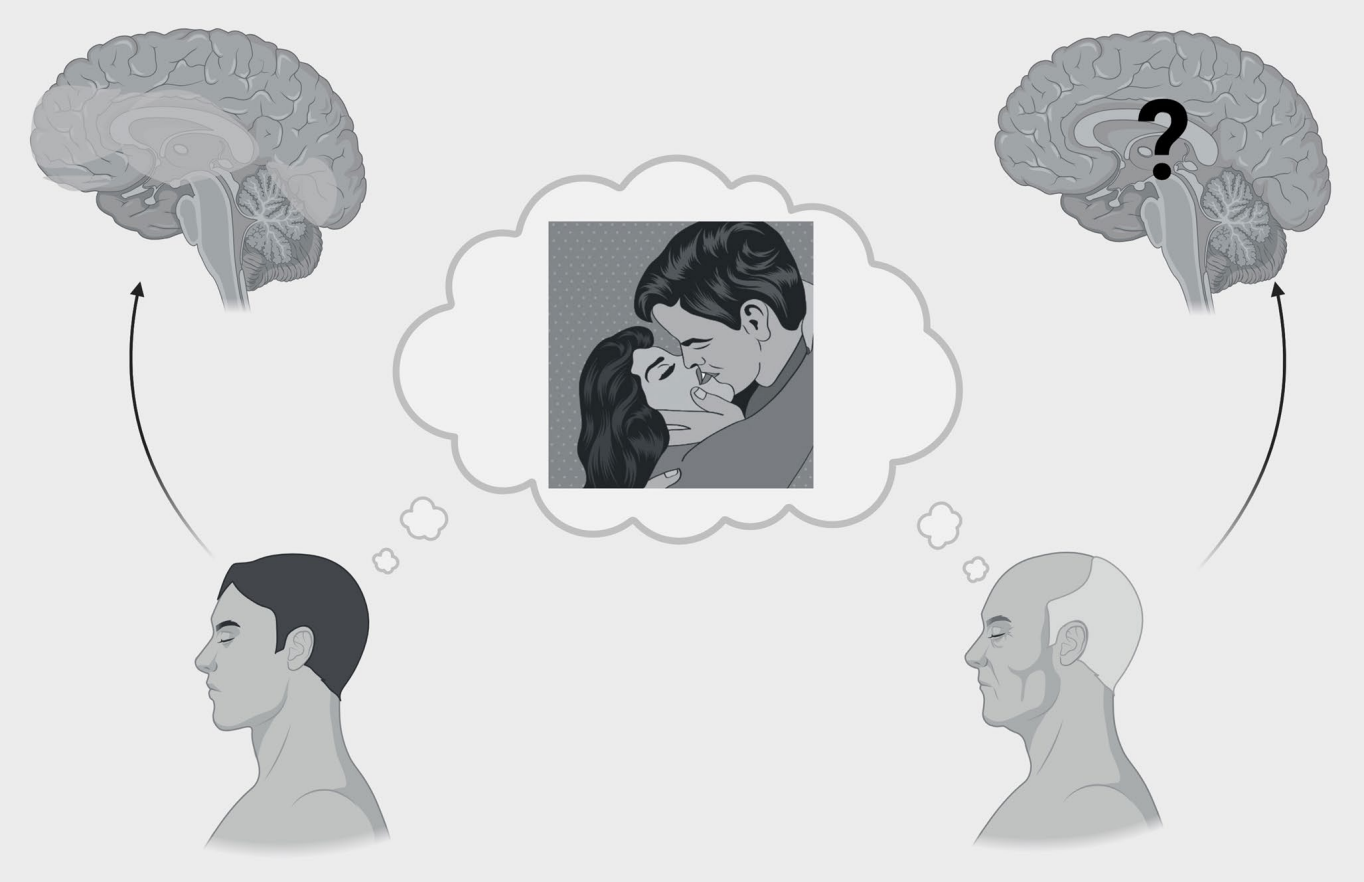
Representation of the need of focusing on older adults, whose sexuality is often neglected. This image does not exhaustively represent all brain areas involved in sexuality, nor the complexity of human sexual preferences and attitudes

This perspective aims to focus on the neuropsychology and psychophysiology of older adults’ sexuality (see Fig. 1), suggesting future research directions and highlighting the importance of this topic. In fact, sexuality is a crucial factor in individuals’ well-being. Smith et al., [4] showed that sexual activity is related with a release of endorphins which contributes to higher levels of happiness. Despite this, the sexuality of older adults is often ignored, with negative stereotypes associated with it. This results in a vicious cycle where this stigmatising perception creates a self-fulfilling prophecy: older adults might refrain from sexual expressions because they believe it is “inappropriate”. Stigma related to sexuality seems to affect healthcare providers as well leading them to contribute to the silence surrounding sexuality in aging [5]. The silence revolving around this topic may impact the possibility for clinicians to identify cases of elder sexual abuse in individuals with and without neurocognitive disorders [6, 7]. From a forensic neuropsychology perspective, it is crucial to assess the individuals’ cognitive capability of understanding, also according to their cognitive status. Consequently, clinicians should inquire about the sexual lives of older adults to overcome the stigma surrounding this subject [4].

The scientific community is also influenced by this stigma, resulting in a scarcity of research on the neuropsychology of sexuality of older adults [5]. Many research questions are still open: what differences in sexuality may be from a neuropsychological perspective between younger and older adults? If these differences are present, what are the roles of physiology and cognitive abilities of individuals? These gaps in literature can have major implications for the clinical field as well.

### Starting from the four components of sexual arousal: what is known and what we need to know

Sexual arousal appears to play a main role in individuals’ sexual experience. Sexual arousal encompasses four interconnected components: cognitive, emotional, motivational and physiological [8]: (1) The former involves the cognitive labelling and subjective experience in determining the response to a given stimulus as sexual [8]; (2) The motivational aspect involves the processes that guide behaviour towards a sexual goal; (3) The emotional aspect pertains to the specific pleasurable quality of sexual arousal; (4) The physiological aspect involves autonomic and endocrinological responses (such as cardiovascular, and genital changes) associated with sexual arousal [9].

Therefore, sexual cognitive and experiential components (e.g., previous experience and social context) are the basis for the peripheral physiological reaction. The physiological reaction then feedbacks to affect cognitive labeling and subjective experience of the stimuli, resulting in sexual arousal, which in turn has an influence on the intensity of physiological arousal [10].

This perspective focuses on the cognitive and physiological components of sexual arousal. Most research articles on the cognitive, neuropsychology and psychophysiology of sexuality have focused on young people, while only a few articles have addressed older adults’ sexuality. Researchers have mainly used visual sexual stimuli to investigate this topic [5]. Moreover, participants’ cognitive labelling and subjective experience are collected through self-reports and interviews, while physiological responses investigated are heart rate, blood pressure, erection and vaginal vasocongestion.

Focusing on the physiological component is essential to investigate it alongside self-reported perceptions of sexual arousal, as research indicates that these two aspects can sometimes conflict [10]. For example, the study by Sarlo and Buodo [11] shows that men had a clear-cut appetitive response pattern when exposed to visual sexual stimuli depicting their preferred sexual target and they had an aversive response toward their non-preferred sexual stimuli. Women, instead, showed a gender non-specific autonomic activation when exposed to both visual sexual stimuli. Based on the literature discussed, the following research question arises: are these results applicable to older adults as well?

### What do we know about the role of the cognitive dimensions related to sexual arousal?

Focusing on the cognitive dimension of sexual arousal, little is known on the brain areas involved in sexual arousal [8] and these few studies did not include older adult samples. These studies have investigated the brain areas activated when exposed to visual sexual stimuli using different neuroimaging techniques: functional Magnetic Resonance Imaging (fMRI) [12]; Positron Emission Tomography (PET) [8]; and the functional Near-Infrared Spectroscopy (fNIRS) [13]. The results of these studies highlight that both men and women when exposed to visual sexual stimuli showed an activation of the anterior cingulate, medial prefrontal, orbitofrontal, insular and occipitotemporal cortices, in the amygdala and ventral striatum [9]. However, literature shows the brain of both genders respond differently to visual sexual stimuli: men present greater activation in the hypothalamus and amygdala, even when women reported greater sexual arousal [12]. The magnitude of the hypothalamic activation, for men only, is positively correlated with reported levels of sexual arousal [9]. Differences are present also in the reaction that men and women have to visual sexual stimuli depicting the sex that they are attracted to. Men’s brains exposed to visual sexual stimuli depicting the sex to which they are attracted show a neural reaction in regions involved in visual attention, motivation, and genital arousal. This neural reaction is little when they are exposed to the other sex. On the contrary, women exposed to visual sexual stimuli show similar reactions to both types of erotic stimuli [14].

One of the few studies on older adults’ brain areas activated during sexual arousal is the one by Padoani et al., [15]. The authors found an association between sexuality and cognitive functioning. The absence of cognitive impairment was found to be linked to the ability to overcome the taboo and physical and psychological problems that could prevent older adults from expressing sexual behaviour. However, this study presents a limitation: only the Mini-Mental State Examination (MMSE) was used to assess the presence of cognitive impairment. The MMSE is a screening neuropsychological instrument that is neither sensitive nor specific enough to assess cognitive functioning [16]. In a recent study, Hartmans et al., [2] showed that participants who perceived sexuality as important and reported higher levels of sexual satisfaction had better general cognitive functioning, memory, processing speed and fluid intelligence. However, Hartmans et al., [16] highlighted that general cognitive functioning was not correlated with changes in sexual behaviour in patients with dementia. Consequently, the relationship between cognitive functioning and sexuality should be further investigated as some results are in conflict and the literature is scarce. In this regard, other research gaps can be listed, among these: are there differences in brain activation between younger and older adults when exposed to visual sexual stimuli? If cognitive functioning is associated with the importance given to sexuality, are there differences between older adults with normal cognitive functioning and those with cognitive impairment?

## Conclusions and future directions

Taking into consideration the above literature, this perspective stressed the research gaps present on the neuropsychology and psychophysiology of older adults’ sexuality. This way it would be possible to overcome the stigma that has negatively affected the research in this area. Moreover, neurocognitive processes underlying sexuality in healthy older adults would pave the way for exploring what happens in individuals affected by neurocognitive diseases. This would also have implications by improving clinicians’ understanding of the role of sexuality of older adults’ well-being.

## Data Availability

No datasets were generated or analysed during the current study.
